# Immunoglobulin heavy chains in medaka (*Oryzias latipes*)

**DOI:** 10.1186/1471-2148-11-165

**Published:** 2011-06-15

**Authors:** Susana Magadán-Mompó, Christian Sánchez-Espinel, Francisco Gambón-Deza

**Affiliations:** 1 Oceanographic Center of Vigo, Spanish Institute of Oceanography (IEO), Subida a Radio Faro 50, 36390 Vigo, Pontevedra, Spain; 2 Shared Unit of Immunology, University of Vigo - Vigo University Hospital Complex (Hospital Meixoeiro), Edificio de Ciencias Experimentales, Rua das Abeleiras, Campus As LagoasMarcosende, Vigo 36310, Pontevedra, Spain; 3 Unidad de Inmunología, Hospital do Meixoeiro, Servizo Galego de Saude (SERGAS), Carretera de Madrid s/n, Vigo 36210, Pontevedra, Spain

## Abstract

**Background:**

Bony fish present an immunological system, which evolved independently from those of animals that migrated to land 400 million years ago. The publication of whole genome sequences and the availability of several cDNA libraries for medaka (*Oryzias latipes*) permitted us to perform a thorough analysis of immunoglobulin heavy chains present in this teleost.

**Results:**

We identified IgM and IgD coding ESTs, mainly in spleen, kidney and gills using published cDNA libraries but we did not find any sequence that coded for IgT or other heavy chain isotypes described in fish. The IgM - ESTs corresponded with the secreted and membrane forms and surprisingly, the latter form only presented two constant heavy chain domains. This is the first time that this short form of membrane IgM is described in a teleost. It is different from that identified in Notothenioid teleost because it does not present the typical splicing pattern of membrane IgM. The identified IgD-ESTs only present membrane transcripts, with Cμ1 and five Cδ exons. Furthermore, there are ESTs with sequences that do not have any VH which disrupt open reading frames.

A scan of the medaka genome using transcripts and genomic short reads resulted in five zones within a region on chromosome 8 with Cμ and Cδ exons. Some of these exons do not form part of antibodies and were at times interspersed, suggesting a recombination process between zones. An analysis of the ESTs confirmed that no antibodies are expressed from zone 3.

**Conclusions:**

Our results suggest that the IGH locus duplication is very common among teleosts, wherein the existence of a recombination process explains the sequence homology between them.

## Background

Genome information of vertebrates is rapidly becoming available thanks to several full vertebrate genome projects. Such information is very useful for comparative and evolutionary biologists. Comparative genomic studies are helping to discover evolutionary mechanisms that underlie diversification of organisms [1,2]. Therefore, information obtained from genomes is of great use for understanding the genetic basis of antibody diversity and the evolutionary divergences of the immunoglobulin locus in vertebrates [3]. Immunoglobulin loci are organised into two main types called: "cluster" and "translocon". Cluster type organization is found in both light and heavy chain loci of cartilaginous fish [4,5] There are many independent variable (VH), diversity (D), joining (JH) and constant (CH) segments sets [VH(D)JHCH] along wide areas of the genome. Therefore, diversity in these molecules is generated through synthesis of antibodies from each of these VH-D-JH-CH regions [6,7]. In tetrapods and bony fish, the IGH locus configuration is translocon and it presents some specific characteristics. There are genomic segments for the variable regions of antibody heavy chains (VH) and these are followed by segments that code for: diversity (D), joining (JH), and segments that encode the heavy chain domains (CH). A rearranged VHDJH region spliced to CH segment is needed to generate an antibody [8,9].

It is well established that all fishes have IGHM and other constant chain region genes in the 3' region. Dooley and Flajnik described genes that encoded the IgW (omega immunoglobulin isotype) and IgNAR (New Antigen Receptor) antibodies in the 3' region, for cartilaginous fish [10-12]. Most bony fish belong to the infraclass teleost, where we can find IgM, IgD [13-15] and IgT/IgZ [16]. However, the IgT/IgZ have not been found in catfish [14]. Teleost IgD is an antibody which generally has seven domains and some of these have experienced recent duplications [17]. The IGHZ (of zebrafish) and IGHT (of rainbow trout) correspond to genes that code for antibodies (IgZ and IgT) with four immunoglobulin domains located upstream from the D and JH segments of IGHM. Furthermore, the exons that code for the constant region present their own D and JH segments, and resemble the organization of T cell receptor alpha and delta (TCR α and δ) loci [18]. Other genes for antibodies found at the same location were described later, and may correspond to different forms of the same antibody [17,19].

Another surprising feature found in some teleost IGH loci, such as in stickleback, catfish [14,17] and medaka, is the presence of core block [VH(D)JHCH] duplications in the germline. Such presence is perhaps not widespread in teleosts because they were not found in zebrafish genome [16]. The duplications present a high homology suggesting that they happened recently or perhaps there is a biological mechanism that maintains them.

This article presents a description of the antibodies in medaka, wherein antibody structure was deduced based on genomic and EST data. Five zones or regions that code for constant chain immunoglobulin domains have been found in genome, and each of these regions has exons for IgM and IgD. Medaka (*Oryzias latipes*), catfish (*Ictalurus punctatus*), zebrafish (*Danio rerio*) and stickleback (*Gasterosteus aculeatus*) represent a group of teleosts that have been widely used as animal models in various fields such as biology, medicine, environmental science and fisheries [20,21]. There is ample information on zebrafish, catfish and stickleback immunoglobulin loci but this is the first time that work on medaka immunoglobulins is published.

## Methods

### Fish and sampling

Adult medaka (*Oryzias latipes*, strain HdrR belongs to the Southern Japanese population) specimens were kindly supplied by J. Cerdá (Institute of Marine Sciences of Barcelona, CSIC, and Aquaculture Centre). Fish were killed by overexposure to MS222 (Sigma Chemicals). Head kidney and spleen were removed aseptically and RNA was extracted immediately using the QIAmp RNA kit (QIAGEN) following manufacturer's instructions.

### cDNA preparation, PCR and DNA sequencing

About 5 μg of total RNA was reverse transcripted into cDNA by using QIAGEN One Step RT-PCR kit and priming with 0.5 μM of Cδ6-antisense primer (5'- GGACTGTTGGAGGATTCATGTCTCACA-3') in a total volume of 50 μl.

Amplification of the IgD constant region was performed in a two-step PCR reaction. 5 μl of cDNA reaction mixture was amplified by thermal cycling in a total volume of 25 μl using Cμ1-sense (5'-CATTGACTTTCTCATGGACTCAGGGC-3') combined with Cδ6- antisense primer. Amplification was performed for 30 cycles at 95°C 30s, 65°C for 30s and 72°C for 90s, with a final elongation step at 72°C for 10 min. Due to a very low amplification product obtained from the first PCR, a second round was performed for 20 more cycles using the same primers and conditions. The amplified products were sequenced on an Applied Biosystems 3130 Genetic Analyzer. The Gepard (GEnome PAir -Rapid Dotter) program [22] was used to search for homologues with the genomic sequences and identify the IgD domains.

### Medaka immunoglobulin expression using ESTs databases

Previously identified immunoglobulin constant heavy chain exons from stickleback [17] were used to search homologue sequences in the medaka ESTs database (http://www.shigen.nig.ac.jp/medaka). A total of 11 cDNA libraries generated from different tissues of HdrR-medaka were scanned (Additional file 1). ESTs encoding for IgM and/or IgD were retrieved. The medaka immunoglobulin ESTs can be found grouped into three clusters: a) CLSTF16513, with the 5' sequences encoding IgM and IgD, b) CLSTR12908 with 3' sequences for IgM and c) CLSTR18886 with 3' IgD sequences.

In order to identify the genomic zone or region that corresponds to each EST, an alignment was performed using the Lastz program available at the Galaxy website (http://main.g2.bx.psu.edu/) [23,24]. To confirm results we performed the same analysis using recently released next generation RNA sequences (SRA023697) deposited in the Sequence Read Archive database of the NCBI (http://www.ncbi.nlm.nih.gov/sra). These alignments were visualized using the Tablet - Next Generation Sequence Assembly Visualization software (http://bioinf.scri.ac.uk/tablet/) [25].

### Identification of the IGH locus

The complete genome of *Oryzias latipes *(assembly: HdrR, October 2005; version 56.1i) built in NCBI (http://www.ncbi.nlm.nih.gov) and Ensembl database platforms (http://www.ensembl.org/index.html) was examined to locate antibody genes. Previously published sequences from other IGHM teleost fish were used to identify genomic scaffolds and chromosomes that contained immunoglobulin genes. These sequences (scaffolds 146, 409 and 501, chromosome 8) were retrieved and analysed in detail using the Vector-NTI (Invitrogen). Two scaffolds were not assigned to any chromosome (scaffold 3172 and 1447) but were identified as harboring IGH gene segments and these scaffolds were observed to overlap on 400 nucleotides suggesting that they are contiguous (Additional File 2).

Identification of exons coding for CH domains was performed by aligning genomic sequences with previously published immunoglobulin mRNAs. Limits of unpublished antibodies were deduced following instructions in the software FGNESH (http://www.softberry.com) and Augustus (http://augustus.gobics.de/submission) [26]. Messenger RNA predicted from the gene sequence was compared with O. latipes EST sequences from NCBI and http://www.shigen.nig.ac.jp/medaka, in order to confirm exon ends and analyse gene expression.

The heavy chain variable segments (VH) of medaka were located on the same scaffolds and chromosome. Several criteria were used to identify VH segments, including: a) the presence of recombination signal sequences (RSS) including the canonical "tattattgt" nonamer sequences (allowing 1 or 2 nucleotide mismatches) and corresponding heptamer sequences, b) the presence of AG and and GT splice sides flanking open reading frames, and c) pattern searches for identifying RSS with 23 bp spacers flanking the 3'end of the VH regions. We verified whether the read sequences corresponded to the VH regions [27].

D segments were identified by the presence of RSS 5' and RSS 3' [24]. They were compared with *O. latipes *EST database in order to confirm their expression. The heavy chain joining (JH) segments were located by homology to published JH sequences. This was carried out by comparing a dot plot between published JH sequences and the 5' region of the IGHM (implementing a window of 30 nt and a match of 60%). RSS was used to detect the beginning of the JH exon while the presence of "GTA" was used to determine the end [27].

The immunoglobulin gene nomenclature used to describe the identified genes followed the guidelines of the international ImMunoGeneTics Information System (http://imgt.org) [28].

In order to resolve occasional mistakes and complete the gaps, all scaffolds retrieved (scaffolds 146, 409 and 501, chromosome 8) were aligned with the recently released genomic new generation sequences (DRA000220), deposited in the Sequence Read Archive database of the NCBI (http://www.ncbi.nlm.nih.gov/sra). The *in silico *analysis was carried out using the available tools at the Galaxy website (http://main.g2.bx.psu.edu/) and visualized with Tablet - Next Generation Sequence Assembly Visualization software (http://bioinf.scri.ac.uk/tablet/).

### Phylogenetic studies

Comparative phylogenetic studies were carried out with the program MEGA5 [29] using the algorithm to perform BLOSSUM alignments. The neighbour-joining and minimum evolution methods were then used to plot the phylogenetic trees (pair-wise deletion, JonesTaylor-Thornton matrix and enter range activated sites (gamma-number 2.5). The veracity of these trees was studied using the above-mentioned method and by executing 1000 replicate bootstrappings.

### GenBank sequences

IgM accession numbers: X83372 *Oncorhynchus mykiss *(rainbow trout), AB2 17624 *Takifugu rubripes *(*Fugu rubripes*), AAQ14862 *Sineperca chuatsi*, AAF69488 *Hippoglossus hippoglossus*, A46538 *Gadus morhua *(Atlantic cod), AAO37747 *Ornithorhynchus anatinus *(platypus) and EU287910 and EU28791 1 *Eublepharis macularius *(leopard gecko). The *G. aculeatus *IGH sequences are in the supplementary information (Additional File 3) [17].

## Results

### Immunoglobulins in medaka

A bioinformatic search of ESTs in the NBRP medaka database (http://www.shigen.nig.ac.jp/medaka/) was carried out in order to determine the kind of antibodies expressed by the teleost medaka. Previously published Cμ, Cδ, Cζ, sequences from *G. aculeatus *[17] were used as queries to identify the ESTs.

A total of 94 EST sequences of IgM and 19 of IgD were identified but we did not find any EST homologues to IgT/Z. Only ESTs with data from both ends (considered full length) were analysed further and are summarized according to their tissue distribution (Table 1). As already reported in other teleosts, IgM and IgD genes are mainly expressed in kidney, gills and spleen. And only a few IgM ESTs were detected in liver, ovarian tissue and brain.

**Table 1:** Tissue distribution for immunoglobulin ESTs

	Gills	Kidney	Spleen	Liver	Ovarian	Brain
***IgM - EST***	10	45	35	1	2	1
***IgD - EST***	1(1)*	11(3)*	7(5)*	0	0	0

* The number in brackets represents the IgD sequences with Cμ1

**Figure 1:**
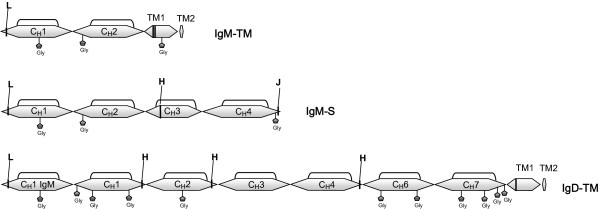
**Immunoglobulin heavy chain constant domains**. The protein structure was deduced from the mRNA coding for transmembrane IgM (IgM-TM), secreted IgM (IgM-S) and transmembrane IgD (IgD-TM) forms. The putative cysteines that establish bonds with light chains (L) and heavy chains (H) to form multimers (J) are shown. Potential glycosylation sites are indicated with Gly.

As shown below, approximately 15% of these ESTs present atypical rearrangement and stop codons in all reading frames. Three immunoglobulin heavy chain forms were found among the viable ESTs. Two of these corresponded to the IgM: transmembrane and secreted forms, and one to transmembrane IgD (Figure 1). Surprisingly, the IgM transmembrane form presented only two CH domains (Cμ1 and Cμ2), TM1 and TM2. The deduced amino acid sequence of medaka IgM showed a cysteine residue in the CH1 domain which is involved in the formation of disulphide bonds with the light chain. We did not find any cysteine in CH1 and CH2 to join the heavy chains; however there were two cysteines in TM1 that may be responsible for covalent heavy chain binding.

The secreted IgM form appears to be similar to those described in other teleostei [14], with four CH domains and a secretory tail. This IgM presents three cysteines for interchain bonds, one in the CH1 domain to establish a disulfide bond with the light chain, another in the CH3 domain to join heavy chains and finally, in the secretory tail, probably to form multimers (Additional File 4).

The study of IgD ESTs permitted us to deduce its structure. This is similar to those described in other teleostei, in which the first constant domain is Cμ1 followed by Cδ1. The Cδ6, Cδ7, TM1 and TM2 domains were present in the ESTs in all cases. All IgD domains expressed could not be described because the forward and reverse ESTs sequences did not overlap. Thus, we decided to perform a RT-PCR of head kidney and spleen mRNA with primers designed for the Cμ1 and Cδ6. A PCR product of approximately 1600 bp was obtained and its sequencing confirmed the presence of Cδ1, Cδ2, Cδ3, Cδ4 and Cδ6 domains (Additional File 5). There was no Cδ5 equivalent in all IgD transcripts sequenced.

### Medaka IGH Genomic organization

**Figure 2:**
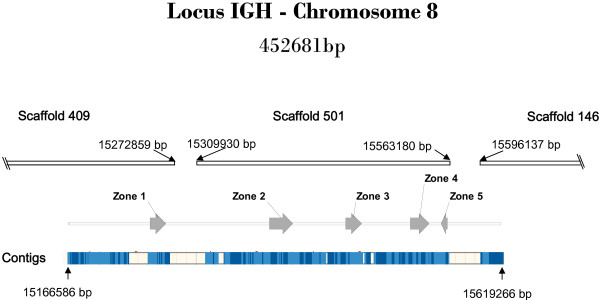
**The IGH locus of *O. latipes***. The image displays the location of scaffolds that encode immunoglobulin heavy chain genes in chromosome 8. Five specific zones can be deduced in which Cμ and Cδ exons are identified. Contigs that cover the IGH locus are represented at the bottom in dark and light blue. The white segments show gaps in the genomic sequence.

Four different sequences of IgM in ESTs were found, suggesting several isotypes or allotypes. These were used to scan the medaka genome and map immunoglobulin heavy chain genes. Several sequences were found on chromosome eight (scaffolds 146, 409 and 501) that cover approximately 450 kb (Figure 2). We also identified two scaffolds that were not assigned to any chromosome (scaffolds 3172 and 1447, with 400 nucleotides overlapped suggesting that they are contiguous) in which several immunoglobulin heavy chain coding exons were annotated.

We were able to elucidate the IGH genomic organization, despite finding several gaps, mainly between scaffolds and contigs, which prevent us from creating a complete contiguous annotation. Furthermore, we were able to complete some gaps and solve several contradictions found between ESTs and genomic sequences, using recently deposited next generation sequence data (DRA000220 and SRA026397) in the Sequence Read Archive Database (http://www.ncbi.nlm.nih.gov/sra).

**Figure 3:**
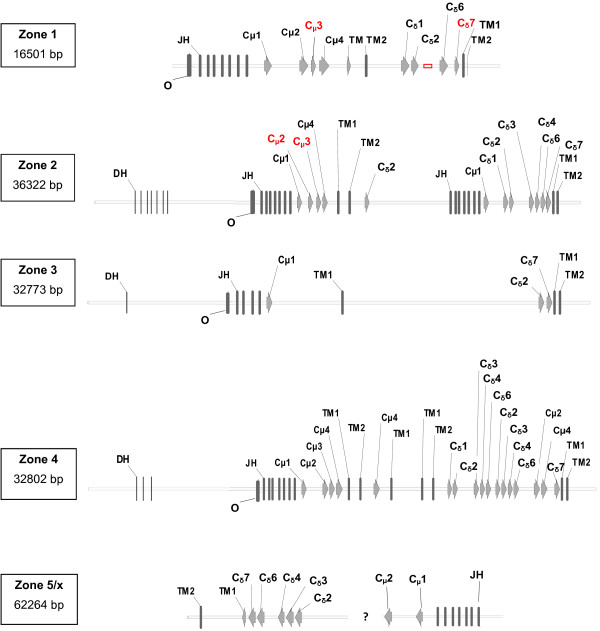
**Detailed representation of five genomic zones identified in *O. latipes *IGH locus**. It shows the D, JH segments, constant (C) and (TM) exons as rectangles or arrowheads. The demarcation of exons was performed by using the computer software (FGNESH and Augustus) and alignment was done with ESTs. The exons and sequences in red were identified or corrected based on the analysis of ESTs and the next generation sequence data (SRA026397 and DRA000220). In zone 5/x the question mark sign (?) indicates the presence of a gap and the possibility that zone x is integrated in zone 5. Zone x presents Cμ sequences belonging to scaffolds 3172 and 1447 and they are not assigned to any chromosome (For more information see Additional file 2).

In order to identify the immunoglobulin heavy chain genes, the genomic scaffolds were divided into 30 Kb segments and analysed using the FGNESH and Augustus software packages [23]. The obtained results were verified through a dot plot and Est2genome (EMBOSS) with available medaka ESTs that had been identified as immunoglobulins. As shown in Figure 2 and 3, the IGH genomic organization is complex, with five specific zones where Cμ and Cδ exons are identified (In Additional File 6 you can find the nucleotide sequences of all zones in GenBank format, we have included a file with VH annotation). It is worthy to note that not all exons are expected to produce an immunoglobulin heavy chain in most zones. Therefore, in order to avoid confusion, instead of naming exons according to their numerical order of appearance in the genomic sequence, we decided to number them according to their orthologous exons defined in other species. The Additional file 7 shows a tree constructed using the aligned medaka CH amino acid sequences and CHs deduced from sequences of the bony fish stickleback (*G. aculeatus*) which supports this criterion for classification.

Overall, Cμ and Cδ exons, D and JH segments (Figure 3) were identified in each of the five genomic zones. No Cδ exons were found and this is consistent with data obtained from ESTs analysis. As indicated in Figure 3 some exons were identified or corrected based on the analysis of ESTs and the next generation sequence data (SRA026397 and DRA000220). VH regions were found between zones (see Additional File 6 and 8).

**Figure 4:**
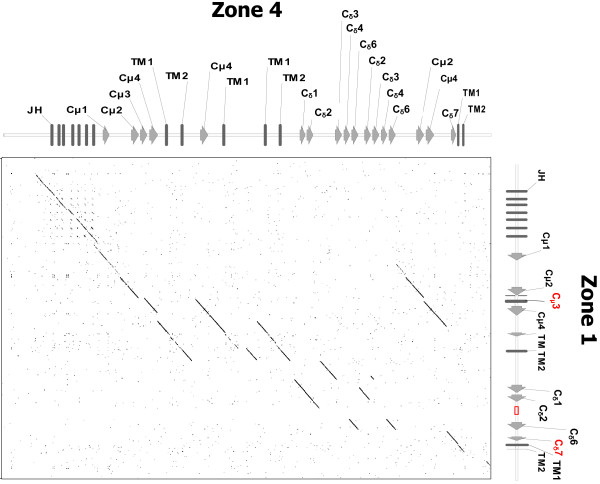
**Gene segment duplications in O. latipes IGH locus**. Dot plot of zone 1 versus zone 4 where the diagonal line corresponds to sequence identity. Segments that are parallel to the diagonal line in the graph indicate duplication. The parameters used for the dot plot diagram were a Window of 30 and 85% identity requirement.

Another important feature is the sequence homology between different zones as shown in Figure 4. A dot plot of zone 1 versus zone 4 genomic sequences elucidated duplications of large and small segments, suggesting a recombination process. The similarity between the sequences of homologous domains and the flanking introns indicates that this process must have taken place recently.

The genomic region designated as zone 1 encodes seven JH segments followed by Cμ1, Cμ2, Cμ3 and Cμ4, and harbors exons that code for a transmembrane and cytoplasm domain. In this zone there are only four Cδ exons (Cδ1, Cδ2, Cδ6 and Cδ7) located 3 kb downstream of the nearest Cμ and are followed by transmembrane and cytoplasm exons. There is a gap between Cδ2 and Cδ6, where there is a high probability of finding the presence of Cδ exons in this first zone.

The remaining zones give us an idea of asymmetric duplications, that is, the presence of Cμ and Cδ exons with a changed configuration. In zone 2, just like in zone 1, the exons Cμ1, Cμ2, Cμ3 (also deduced from EST sequences) and Cμ4 appear after seven D segments and seven JH segments (Figure 3). Exons for transmembrane and cytoplasm domains are also present. At 3' of these exons, we find one Cδ2 exon without any other sequence coding for IgD antibody. Interestingly, about 5 kb downstream, we find D and JH segments followed by Cμ1 and Cδ1- Cδ2- Cδ3- Cδ4- Cδ6 exons again. Therefore, we can differentiate two genomic regions in this zone, namely; zone 2a at 5', and zone 2b at 3'. Both of them have exons to IgM and IgD.

The zone designated as zone 3 seems to be quite disorganized when compared with other zones. As shown in Figure 3, there are very few exons and this suggests that this zone may not generate functional antibodies. Conversely, zone 4 appears to be well structured and presents the highest number of exons. At the 5' region there are four Cμ exons, including Cμ2 with their transmembrane and cytoplasm coding exons (Figure 3). Surprisingly, domain Cμ4 and the transmembrane and cytoplasm exons are found to be duplicated. At the 3' region, there are 10 exons for IgD domains, some of which are repeated (Cδ2, Cδ3, Cδ4 and Cδ6). Between the last Cδ6 and the Cδ7 there are exons that code for IgM (Cμ2 and Cμ4) and finally, we find sequences for transmembrane and cytoplasm IgD domains.

At present, zone 5 is the least resolved genomic region. This is due to the presence of a gap of about 30 kb between scaffold 501 and the 146 junction. The identified sequences, D segments and Cδ exons, are found to be inverted. The IGHM might be missing due to the presence of the gap however; it is very probable that scaffolds 3172 and 1447 belong to the gap because they are not assigned to any chromosome and present sequences for IgM domains (See Figure 3). The above will be taken into account and from now on we will be referred to as zone5/x.

### Correlation between ESTs and genomic sequences

**Figure 5:**
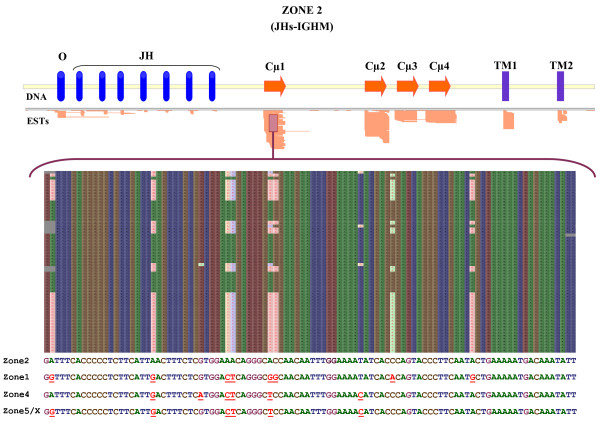
**Confirmation of different genomic zones through ESTs alignment**. The top part of the figure shows the result obtained after aligning medaka immunoglobulin coding ESTs with the region of zone 2 that presents JH segments and the IGHM gene. A higher number of ESTs matches with Cμ1 and Cμ2 is due to their presence in transmembrane and secreted IgM. The bottom part shows details of ESTs alignment with the Cμ1. It includes the specific nucleotide sequence to each zone. Identical nucleotides are shown in the same colour (A: green, C: brown, G: lilac, T: blue) and differences are in red and underlined.

The structure of this multiple locus with a high number of repetitions and with exons situated out of normal locations made us verify this annotation. To do this we aligned the ESTs and DNA short reads (DRA000220) with the different zones of the immunoglobulin locus. For example, Figure 5 shows the alignment of the ESTs with the exon Cμ1 belongs to zone 2. We identified ESTs with specific differences that correspond to each of the Cμ1 sequences found in the different zones, except in zone 3 revealing that it is possible to assign ESTs to concordant gene segments (Figure 5). When aligned with the DNA short reads we found the same results but in this case we were able to detect short reads belonging to zone 3 (Additional file 9).

In addition, the tissue distribution of ESTs from lymphoid tissues (kidney, spleen and gills) was correlated with genomic zones. ESTs were found to be expressed from 1, 2, 4 and 5/x zones (Table 2).

**Table 2:** IgM coding ESTs expressed by each genomic zone

	Zone 1	Zone 2	Zone 3	Zone 4	Zone 5/x
IgM-S*	21	26	0	5	2

IgM-TM*	13	6	0	11	2

* IgM-S: Secreted Immunoglobulin M

** IgM-TM: Transmembrane Immunoglobulin M

On the subject of ESTs coding for IgM, we identified a total of 34 ESTs expressed from zone 1, where 21 corresponded to the secreted form and 13 to the membrane form (Table 2). Thirty-two ESTs were assigned to zone 2, with membrane (6 ESTs) and secreted (26 ESTs) forms. Eleven IgM membrane and five IgM secreted coding ESTs belonged to zone 4. Only 8 IgM ESTs (4 membrane and 4 secreted) were found to be expressed from zone 4 and, as expected due to its disorganized genomic structure, no EST from zone 3 was detected.

**Figure 6:**
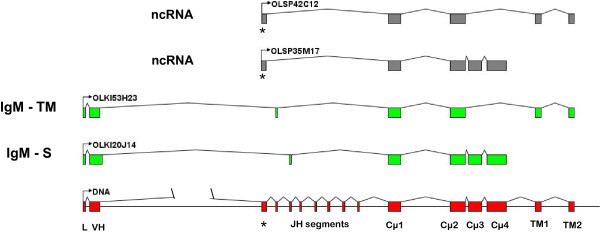
**Schematic representation of four IgM ESTs and their relationship with the genomic sequence**. Examples of the different RNA splicing found in EST database are indicated. Transcripts for secreted form of IgM (OLKI20J14) with four Cμ domains and for transmembrane IgM (OLKI53H23) with two Cμ and transmembrane domains are shown. Non coding transcripts (ncRNA) were found too, these included a sequence at the 5'JH region named exon 0 (*) and have stop codons in the three reading frames.

It is worthy to note that approximately 15% of these ESTs (for example the 6 ESTs assigned to zone 4) do not show the classical rearrangement (VH-D-JH-CH) and are expressed without VH segment. Joining in most of these rearrangements takes place between an exon situated at 3' of JH segments and Cμ1. This 3'-JH exon (named Exon 0) has 100 bp and presents stop codons in the 3 ORF (Figure 6). This exon is in zone 1, 2 and 3 and not in 4 and 5/x. Not all mRNAs without VH have this exon 0, for example, 6 ESTs assigned to zone 4.

**Figure 7:**
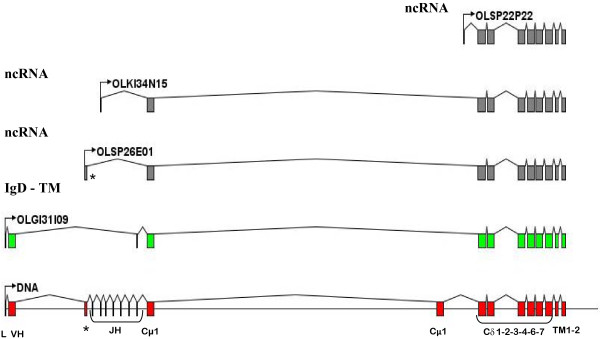
**Schematic representation of the splicing mechanisms generating IgD and their relationship with the genomic sequence**. Examples of the four RNA splicing found in EST database are indicated. All ESTs are membrane antibodies. Only OLGI31I09 presents all exons to code for a chimeric transmembrane IgD with a Cμ1 domain. Furthermore, mRNA without variable region (OLSP26E01, OLKI34N15 and OLSP22P22) were found, all of them have stop codons in all reading frames.

Just like in other fishes, we identified 19 ESTs coding for the membrane IgD form. However, we were unable to unequivocally establish the distribution of these ESTs in each genomic zone because the different Cδ genomic sequences were very similar. Moreover, only four ESTs corresponded to the typical chimeric transcripts observed in teleosts, (utilizing exon Cμ1) and presented the typical VH-D-JH rearrangement. Other IgD transcripts found presented an atypical rearrangement with Cδ domains and diversity in the 5' region, some examples are shown in (Figure 7) where olsp26e01 presents the exon 0 spliced to Cμ1, olki34n15 lacks VH and olsp22p22 includes genomic sequences adjacent to Cδ1.

## Discussion

The analysis of cDNA libraries obtained from different tissues permitted the identification of Ig exons expressed in medaka (*Oryzias latipes*). ESTs coding for IgM and IgD were identified but no expression or genomic data was found for other isotypes in medaka.

In mammals, the production of secreted and membrane IgM forms involves alternative splicing. The transmembrane form is originated through a cryptic splice site located within Cμ4 that have the acceptor site at 3' of the TM1 exon [30]. This pattern is manifested in Xenopus and cartilaginous fish too [31-33]. However, in teleosts the transmembrane IgM is comprised of the first three exons (Cμ1, Cμ2 and Cμ3) plus the transmembrane and cytoplasm exons [34,35]. The splicing pattern of IgM appears consistent with exceptions only in a few species, for example, membrane IgM chains with different number of Cμ domains have been described in ancient fishes [30,36], in which we observe the general rule followed in teleost fishes, Cμ3 - TM, as well as the mammalian pathway, Cμ4 - TM. However, in Siberian Sturgeon, the splicing pattern can result in a transmembrane immunoglobulin with four, two and half, or only one Cμ domain [37]. Notothenioid teleosts membrane IgM transcripts likewise lack the Cμ3, and the Cμ2 is spliced to two short exons (RA and RB) creating an elongated extracellular membrane-proximal domain [38]. Nevertheless, the splicing observed in medaka occurs between the end Cμ2 and TM1 and produces a membrane antigen receptor of only two constant immunoglobulin domains. This is the first time that in a typical teleost is described to have a short transmembrane IgM and indicates that other teleosts may have evolved to exhibit considerable diversity in IgM splicing. Such diversity may be due to a selection process or due to "genomic configurations" that led to the modification of the splicing machinery.

The medaka IgD transcripts studied correspond to the membrane form and, just as in other teleosts, are chimeric, with the inclusion of Cμ1 and six Cδ exons. The Cμ1 exon permits covalent association with light chains, this kind of splicing (Cμ1 to Cδ exon) is not only restricted to teleosts as it has recently been described in porcine IgD transcripts. One interesting feature is that medaka transcripts lack the canonical Cδ5 exon and this finding is confirmed in the genomic sequence, where IGHD loci seem to have been subjected to dramatic recombination events leading to loss of the Cδ5 exon. A high diversity in IGHD genes has been described in teleosts [39,40]. Seven Cδ domains comprise the backbone of many bony fish delta chains, wherein a wide range of domain organization within fish lineages is observed. In the Japanese flounder (*Paralichthys olivaceus*) [41] and stickleback (*Gasterosteus aculeatus*) [17], the IGHD locus consists of the Cδ1-Cδ2-Cδ3-Cδ4-Cδ5-Cδ6-Cδ7-TM1-TM2 exons, in which the homology of domains CH2-CH5, CH3-CH6 and CH4-CH7 suggests that Cδ2-Cδ3-Cδ4 duplicated to generate Cδ5-Cδ6-Cδ7 [17,39]. However, in Atlantic salmon (*Salmo salar*), grass carp (*Ctenopharyngodon idella*) and catfish (*Ictallurus punctatus*) a duplication of Cδ2-Cδ3-Cδ4 has been described [15,34,42]. In Atlantic cod (*Gadus morhua*) the IGHD locus has undergone rearrangement events leading to the loss of Cδ3, Cδ4, Cδ5 and Cδ6 exons with a tandem duplication of the Cδ1-Cδ2 region. It appears that diversification of IgD may be due to germline changes that are species specific rather than due to different splicing pattern as described in IgM. Therefore, only in sharks partly of IgD, like W heavy chain, is diversified through alternative splicing. Further studies are needed to understand the reason for this phenomenon and the biological/evolutive meaning of both mechanisms to generate antibody diversity.

Analysis of ESTs showed that there were atypical IgM and IgD transcripts (approx. 15%), which had stop codons interrupting the reading frames. Most of them lacked the VH region and contained a genomic sequence, named exon 0, at the 5' location, which is spliced directly to the constant exons. It is common to find sterile transcripts from light chain loci in teleosts, and these may be associated to the high frequency of enhancers in the IgL loci of bony fishes [43,44]. Recently, unusual IgD transcripts have also been described in *Salmo salar *[45], wherein the VH and JH sequences are not obvious and include genomic sequences. In catfish [13], in which the Cδ1 is directly spliced to leader exon, which was shown to be functional and capable of mediating secretion of IgD from catfish B cells. The authors suggest the possibility that this secreted IgD functions as a pattern-recognition molecule. These results observed in the several teleost species suggest an evolutive and functional role for non-traditional VHDJH rearrangement and needs to be studied in the future. In medaka, the splicing between exon 0 and the rest of the exons indicate that all components of the immunoglobulin heavy chain, except the VH region, are needed for a specific process in the teleosts.

The ESTs encoding medaka IgM present differences in their Cμ nucleotide sequences, suggesting a duplicated IGH locus in medaka. Therefore, when we scanned the medaka genome with these ESTs we found a very complex locus, with five tandem duplicated Cμ. and Cδ genes separated by VH, D and JH segments. In other fishes we can find duplicated IGH loci, like in *I. punctatus, G. aculeatus, S.salar *[17,19,40,45] or, like in zebrafish (*Danio rerio*), only one IGH copy [46]. Duplicated segments in medaka showed a high DNA level homology for exons and introns. The most probable explanation is such duplications occurred recently and take place frequently. In the future, it would be of interest to identify the mechanism responsible for this genetic exchange. Preliminary data indicates the presence of short repeated sequences (SRS)s at the beginning of duplications suggesting their involvement in such exchange processes (data not shown).

The current medaka whole genome sequence draft presents a number of gaps that do not permit exact delineation of gene configuration. Just like in the case of other vertebrates, the IGH locus has regions that are quite difficult to sequence, due to the frequent presence of SRS. Additionally, the analysis of the medaka germline IGH locus gave rise to uncertainties which on the one hand suggested the lack of Cμ3 and on the other identified Cδ7 as a pseudogene. The database of ESTs and the recently released next generation sequence data from Illumina enabled us to confirm the presence of Cμ3 and Cδ7 as functional exons. However, the high sequence homology between the duplicated segments prevented us from providing a gap-free IGH locus annotation using this additional information.

Despite the medaka IGH locus having many genes, no genes for IgT/Z have been so far identified as has been the case in catfish [14]. Furthermore, we found exons and even entire zones (Ex. zone 3) that were not expressed. It is difficult to explain the evolutive significance of the presence of exons, which are predicted to be functional (without stop codon or any other alteration in their sequences) but are not going to be expressed. Perhaps the screening and sequencing of EST libraries was not sensitive enough to detect mRNA in low concentrations. However, it seems improbable that zone 3 could generate a functional antibody. A possible explanation for sequence maintenance would be its relationship with the genetic locus structure itself. The high number of recombinations may determine that the predicted functional exons cannot generate antibodies in the medaka strain studied, even though antibodies were expressed by non-homologue recombination in other medaka fish. In order to verify this hypothesis, the sequencing of these loci in other fish strains of the same species should give us different haplotypes.

IGH locus duplications appear to be common in teleost fishes and should be favoured by natural selection. These observations indicate that these duplications may have arisen in a common ancestor teleost or are due to independent gene duplications that occurred in each specie through their specific phylogenetic history. The fact that many teleosts appear to harbor duplications may support the first hypothesis, however there are also data that suggest an independent evolution in different lineages. The high homology between different zones of the IGH locus (as exons as introns) indicates recent duplications processes. However, if they took place a long time ago, then recombinations events would be required to explain sequence maintenance. In medaka, such duplications and recombinations could explain the presence of immunoglobulin constant exons in germline IGH locus, which are apparently functional but are not expressed. The same reasoning can be applied in the case of VH segments, to explain high homology between members of the same family. Thus, all chromosome segments that contain the IGH locus would be subjected to such duplication and recombination processes.

Duplicated genes have been identified in many teleostean fishes and it has been suggested that species diversity might be related to large-scale independent gene duplications or to whole genome duplication in an ancient teleost [47,48]. In the case of IGH locus several particular issues remain to be explored. The mechanism known as allelic exclusion prevents the production of more than one specificity in a single lymphoid cell, only one rearrangement product of immunoglobulin is transcribed and translates [49]. Studies of the allelic exclusion of immunoglobulin genes have been performed in species in which a single IGH locus undergoes somatic rearrangement through the lymphocyte development. However, the mechanisms by which teleosts such as medaka, stickleback, catfish, salmo with several IGH locus duplications can exhibit allelic exclusion remains unknown. In medaka, there are at least four IGH duplications that are functional. This means that one cell has the possibility to produce four heavy chains at the same time and therefore could deviate substantially from the clonal selection theory. Eason et al. [50], identified different productive gene transcripts in isolated single peripheral blood lymphocytes from cartilaginous fish (*Raja eglanteria*), indicating the possibility of simultaneous immunoglobulin heavy chains expression from multiple different IGH loci in fishes. In cartilaginous fishes, the IGH locus is arranged in multiple independent clusters, thus indicating that the regulation of immunoglobulin expression could be very different from teleost fishes in which the IGH locus is typically in translocon configuration. The fundamental question regarding the establishment and maintenance of haplotype exclusion in a complex multi-cluster- translocon system such as found in medaka IGH locus remains unanswered today.

Further studies are required to a) understand whether IGH locus duplications involve additional biological mechanisms in the immune system and b) to gauge the potential evolutive advantages of such configurations to the generation of immunoglobulin diversity in these species.

## Conclusions

The present study shows the genomic organization of the IGH locus in medaka that has genes for IgM and IgD however, no Cτ genes have been identified upstream of the Cμ region. This IGH locus is very complex, with five duplications that present high homology, being four of them functional. Our results suggest that the IGH locus duplication is very common among teleosts, wherein the existence of a recombination process explains the sequence homology between them.

## Authors' contributions

SMM and FGD designed the study, carried out gene annotation, sequences analysis and molecular labwork. CSE participated in sequences alignment and gene annotation. All authors participated in writing the manuscript and read and approved the final version.

## Supplementary Material

Additional file 1 **List of medaka full length cDNA libraries**. The table Table 1S shows all cDNA libraries obtained from different medaka tissues that were analysed to perform this study. The number of cDNA sequences of each library is indicated. Click here for file

Additional file 2 **A detailed representation of genomic zone x identified in *O. latipes *IGH locus**. It shows the D, JH segments, constant (C) exons as rectangles or arrowheads. The image displays the location of scaffolds in which these segments were described. Click here for file

Additional file 3 **IGH locus with location of the VH genes from *G.aculeatus***. In this file you can find the G. aculeatus IGH locus sequence with the annotation of the different genic segments. All VH segments present identifiable leader and RSS sequences. This file can be visualized with Vector-NTI program available in http://www.invitrogen.com. Click here for file

Additional file 4 **Amino acid sequence alignment of medaka IgM**. Alignment is made with the nearest sequences found in Genbank. Canonical cysteines are linked by a line. The first cysteine is marked with an arrow and corresponds to the cysteine required for binding to light chains. Other interesting non-canonical cysteines are also marked with an arrow. Click here for file

Additional file 5 **RT-PCR analysis of medaka IgD**. Total RNA was obtained from head kidney (HK) and spleen (SP) and RT-PCR was performed using specific primers for Cμ1 and Cδ6. B) IgD domains identification through dot plot of IgD coding cDNA and genomic zone 4 sequence. Click here for file

Additional file 6 **Medaka germline nucleotide sequences**. In this file you can find nucleotide sequences of different zones described in the medaka IGH locus. The IGHVs file contains the annotation of all VHs. All files can be visualized with Vector-NTI program available in http://www.invitrogen.com. Click here for file

Additional file 7 **Phylogenetic trees**. This figure show unrooted phylogenetic trees made with domains of *O. latipes *and *G. aculeatus *δ left) and μ (right) chains. MEGA4 software, minimum evolution algorithm and JTT matrix were used to draw the tree. Differences by site are activated with gamma parameter 2.5. * The *G. aculeatus *Cδ5 does not present any orthologous exon in medaka IGH locus. Click here for file

Additional file 8 **IGH locus diagram with location of the VH genes from *O. latipes***. All genes were localized as indicated in material and methods (top left). Alignment of aminoacid sequences deduced from VH genes are at the bottom. The alignment was performed and scored according to recommendations of the IMGT. Naming was done using nomenclature proposed by the same organization. Phylogenetic tree of the VH regions from *O. latipes *is shown on the top right hand side. MEGA4 software, minimum evolution algorithm and JTT matrix were used to draw the tree. Differences by site are activated with gamma parameter 2.5. Click here for file

Additional file 9 **Confirmation of different genomic zones through genomic short reads alignment**. This file shows the result obtained after aligning medaka genomic short reads (DRA000220) with the region of zone 1 that presents JH segments and the IGHM gene. Alignment details of short reads with the Cμ1 are indicated. It includes the specific nucleotide sequence to each zone. Identical nucleotides are shown in the same colour (A: green, C: brown, G: lilac, T: blue) and differences are in red and underlined. Click here for file
