# Supplementary material for: Immunoglobulin heavy chains in medaka (Oryzias latipes)
Source: BMC Evol Biol. 2011 Jun 15;11:165. doi: 10.1186/1471-2148-11-165 (PMC3141427; doi:10.1186/1471-2148-11-165)
Supplement: Additional file 1 — List of medaka full length cDNA libraries. The table Table 1S shows all cDNA libraries obtained from different medaka tissues that were analysed to perform this study. The number of cDNA sequences of each library is indicated. [file 1471-2148-11-165-S1.PDF]

Table S1 : Medaka full length cDNA libraries (http://www.shigen.nig.ac.jp)

Library	Tissue	5´Sequences	3´ Sequences	Total Sequences
**olea**	Embryo	22258	22215	44473
**oleb**	Embryo	23134	23127	46261
**olec**	Larvae	23000	23138	46138
**olvl**	Liver	18867	18978	37845
**olli**	Liver	23315	23534	46849
**olbr**	Brain	22582	22442	45024
**olgi**	Gill	22803	22826	45269
**olki**	Kidney	23150	22979	46129
**olsp**	Spleen	23402	23433	46835
**olte**	Testis	24301	24342	48643
**olova**	Ovary	22934	23184	46118
