# Supplementary figures and images for: Immunoglobulin heavy chains in medaka (Oryzias latipes)
Source: BMC Evol Biol. 2011 Jun 15;11:165. doi: 10.1186/1471-2148-11-165 (PMC3141427; doi:10.1186/1471-2148-11-165)

# Zone X

41751 bp

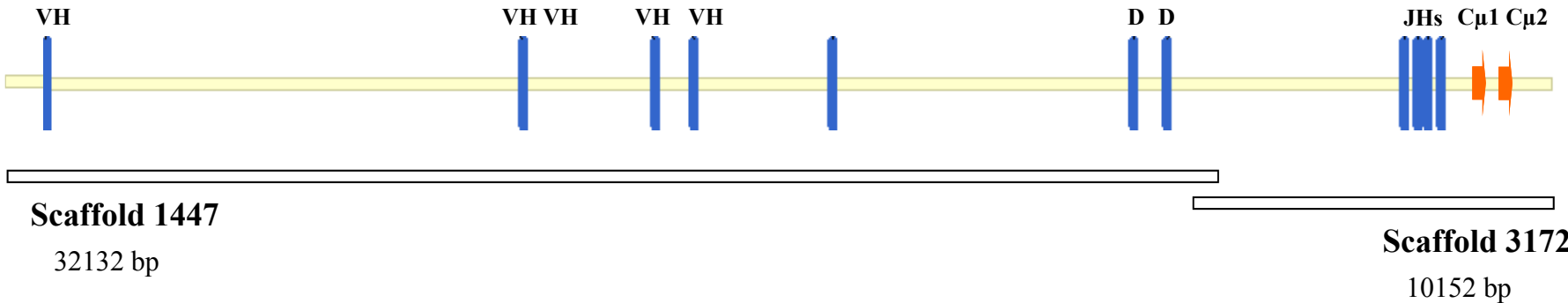

Supplement: Additional file 2 — A detailed representation of genomic zone x identified in O. latipes IGH locus. It shows the D, JH segments, constant (C) exons as rectangles or arrowheads. The image displays the location of scaffolds in which these segments were described. [file 1471-2148-11-165-S2.PDF]

**A**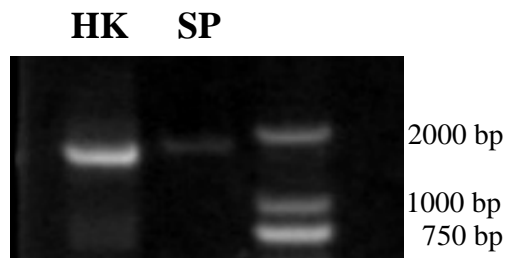**B**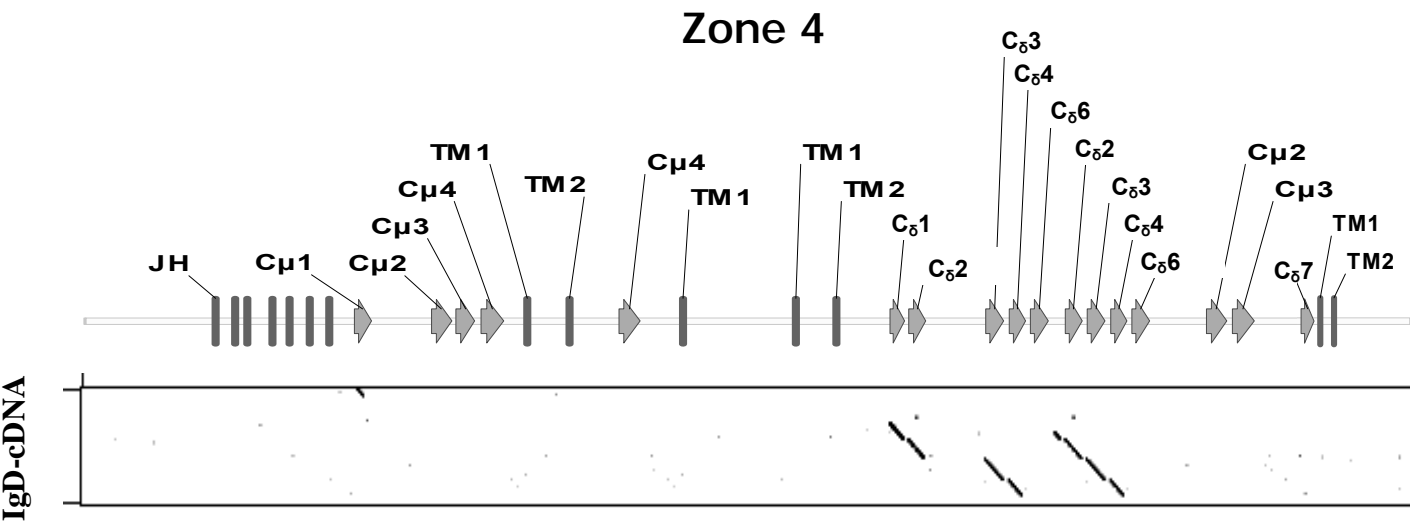

Supplement: Additional file 5 — RT-PCR analysis of medaka IgD. Total RNA was obtained from head kidney (HK) and spleen (SP) and RT-PCR was performed using specific primers for Cμ1 and Cδ6. B) IgD domains identification through dot plot of IgD coding cDNA and genomic zone 4 sequence. [file 1471-2148-11-165-S5.PDF]

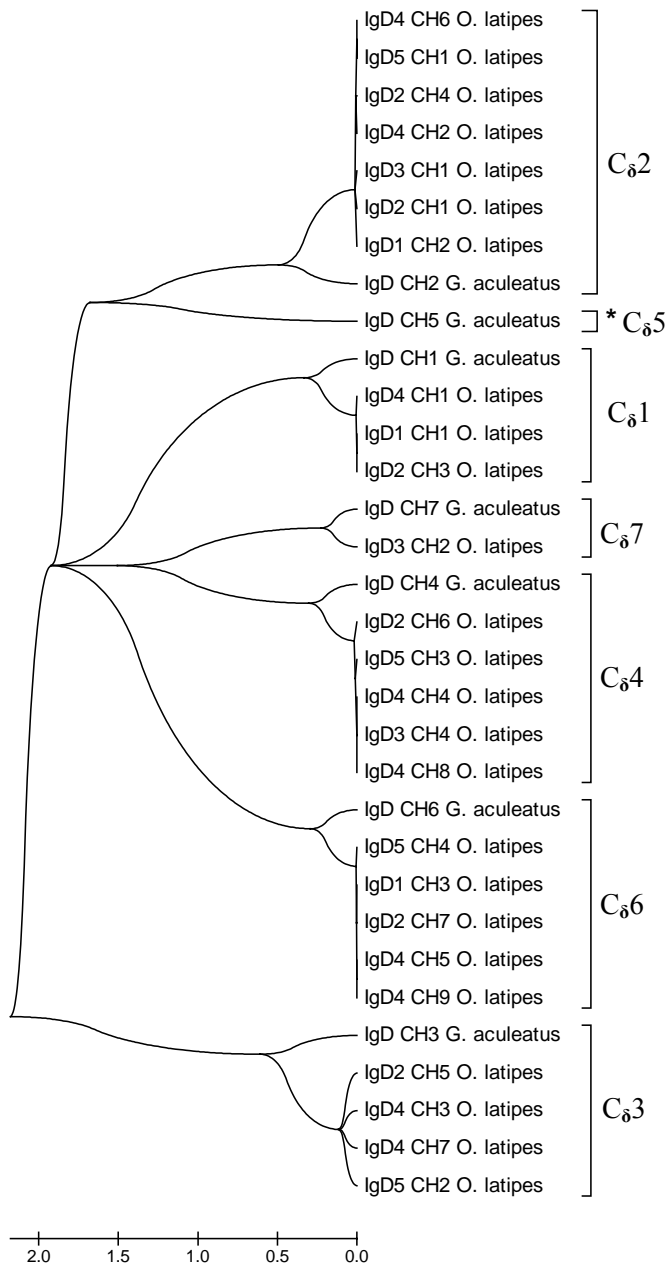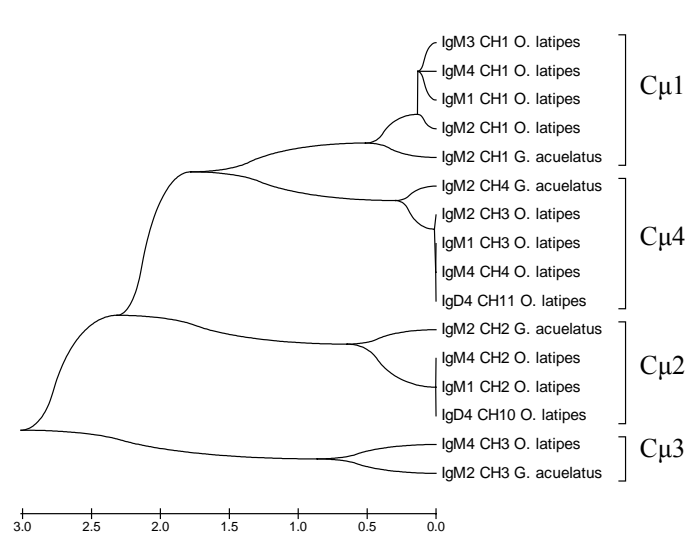

Supplement: Additional file 7 — Phylogenetic trees. This figure show unrooted phylogenetic trees made with domains of O. latipes and G. aculeatus δ left) and μ (right) chains. MEGA4 software, minimum evolution algorithm and JTT matrix were used to draw the tree. Differences by site are activated with gamma parameter 2.5. * The G. aculeatus Cδ5 does not present any orthologous exon in medaka IGH locus. [file 1471-2148-11-165-S7.PDF]

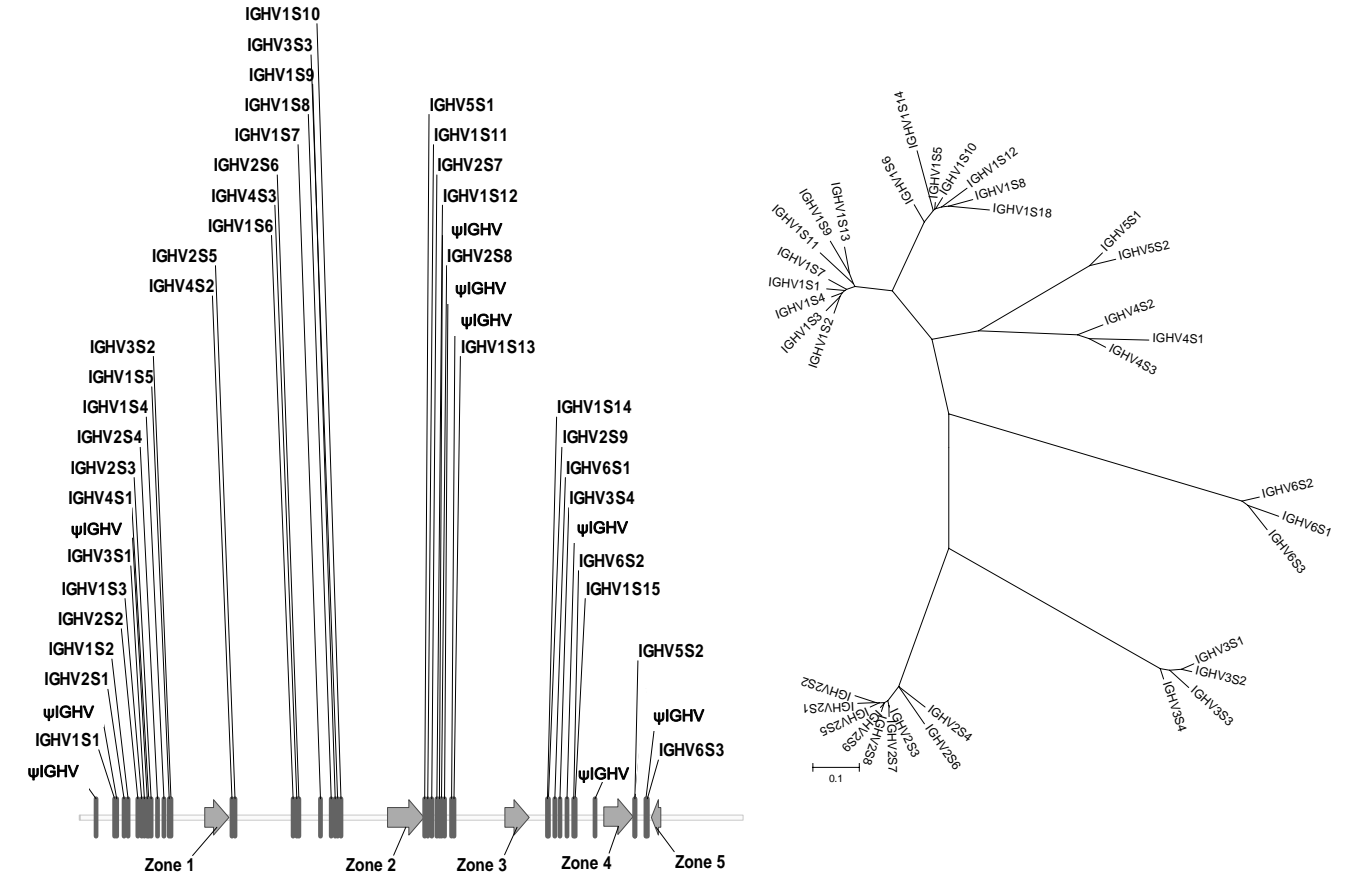

| IGHV gene | FR1 (1-28) |    |    |    |    |    |    | CDR1 (29-36) | FR2 (37-53) |    |     |  | CDR2 (54-62) | FR3 (63-100) |  |  |  |  |  | CDR3 (101-103) |  |  |  |  |  |  |  |  |  |  |  |  |  |  |  |  |  |  |  |  |  |  |  |  |  |  |  |  |  |  |  |  |  |  |  |  |  |  |  |  |  |  |  |  |  |  |  |  |  |  |  |  |  |  |  |  |  |  |  |  |  |  |  |  |  |  |  |  |  |  |  |  |  |  |  |  |  |  |  |  |  |  |  |  |  |  |  |  |  |  |  |  |  |  |  |  |  |  |  |  |  |  |  |  |  |  |  |  |  |  |  |  |  |  |  |  |  |  |  |  |  |  |  |  |  |  |  |  |  |  |  |  |  |  |  |  |  |  |  |  |  |  |  |  |  |  |  |  |  |  |  |  |  |  |  |  |  |  |  |  |  |  |  |  |  |  |  |  |  |  |  |  |  |  |  |  |  |  |  |  |  |  |  |  |  |  |  |  |  |  |  |  |  |  |  |  |  |  |  |  |  |  |  |  |  |  |  |  |  |  |  |  |  |  |  |  |  |  |  |  |  |  |  |  |  |  |  |  |  |  |  |  |  |  |  |  |  |  |  |  |  |  |  |  |  |  |  |  |  |  |  |  |  |  |  |  |  |  |  |  |  |  |  |  |  |  |  |  |  |  |  |  |  |  |  |  |  |  |  |  |  |  |  |  |  |  |  |  |  |  |  |  |  |  |  |  |  |  |  |  |  |  |  |  |  |  |  |  |  |  |  |  |  |  |  |  |  |  |  |  |  |  |  |  |  |  |  |  |  |  |  |  |  |  |  |  |  |  |  |  |  |  |  |  |  |  |  |  |  |  |  |  |  |  |  |  |  |  |  |  |  |  |  |  |  |  |  |  |  |  |  |  |  |  |  |  |  |  |  |  |  |  |  |  |  |  |  |  |  |  |  |  |  |  |  |  |  |  |  |  |  |  |  |  |  |  |  |  |  |  |  |  |  |  |  |  |  |  |  |  |  |  |  |  |  |  |  |  |  |  |  |  |  |  |  |  |  |  |  |  |  |  |  |  |  |  |  |  |  |  |  |  |  |  |  |  |  |  |  |  |  |  |  |  |  |  |  |  |  |  |  |  |  |  |  |  |  |  |  |  |  |  |  |  |  |  |  |  |  |  |  |  |  |  |  |  |  |  |  |  |  |  |  |  |  |  |  |  |  |  |  |  |  |  |  |  |  |  |  |  |  |  |  |  |  |  |  |  |  |  |  |  |  |  |  |  |  |  |  |  |  |  |  |  |  |  |  |  |  |  |  |  |  |  |  |  |  |  |  |  |  |  |  |  |  |  |  |  |  |  |  |  |  |  |  |  |  |  |  |  |  |  |  |  |  |  |  |  |  |  |  |  |  |  |  |  |  |  |  |  |  |  |  |  |  |  |  |  |  |  |  |  |  |  |  |  |  |  |  |  |  |  |  |  |  |  |  |  |  |  |  |  |  |  |  |  |  |  |  |  |  |  |  |  |  |  |  |  |  |  |  |  |  |  |  |  |  |  |  |  |  |  |  |  |  |  |  |  |  |  |  |  |  |  |  |  |  |  |  |  |  |  |  |  |  |  |  |  |  |  |  |  |  |  |  |  |  |  |  |  |  |  |  |  |  |  |  |  |  |  |  |  |  |  |  |  |  |  |  |  |  |  |  |  |  |  |  |  |  |  |  |  |  |  |  |  |  |  |  |  |  |  |  |  |  |  |  |  |  |  |  |  |  |  |  |  |  |  |  |  |  |  |  |  |  |  |  |  |  |  |  |  |  |  |  |  |  |  |  |  |  |  |  |  |  |  |  |  |  |  |  |  |  |  |  |  |  |  |  |  |  |  |  |  |  |  |  |  |  |  |  |  |  |  |  |  |  |  |  |  |  |  |  |  |  |  |  |  |  |  |  |  |  |  |  |  |  |  |  |  |  |  |  |  |  |  |  |  |  |  |  |  |  |  |  |  |  |  |  |  |  |  |  |  |  |  |  |  |  |  |  |  |  |  |  |  |  |  |  |  |  |  |  |  |  |  |  |  |  |  |  |  |  |  |  |  |  |  |  |  |  |  |  |  |  |  |  |  |  |  |  |  |  |  |  |  |  |  |  |  |  |  |  |  |  |  |  |  |  |  |  |  |  |  |  |  |  |  |  |  |  |  |  |  |  |  |  |  |  |  |  |  |  |  |  |  |  |  |  |  |  |  |  |  |  |  |  |  |  |  |  |  |  |  |  |  |  |  |  |  |  |  |  |  |  |  |  |  |  |  |  |  |  |  |  |  |  |  |  |  |  |  |  |  |  |  |  |  |  |  |  |  |  |  |  |  |  |  |  |  |  |  |  |  |  |  |  |  |  |  |  |  |  |  |  |  |  |  |  |  |  |  |  |  |  |  |  |  |  |  |  |  |  |  |  |  |  |  |  |  |  |  |  |  |  |  |  |  |  |  |  |  |  |  |  |  |  |  |  |  |  |  |  |  |  |  |  |  |  |  |  |  |  |  |  |  |  |  |  |  |  |  |  |  |  |  |  |  |  |  |  |  |  |  |  |  |  |  |  |  |  |  |  |  |  |  |  |  |  |  |  |  |  |  |  |  |  |  |  |  |  |  |  |  |  |  |  |  |  |  |  |  |  |  |  |  |  |  |  |  |  |  |  |  |  |  |  |  |  |  |  |  |  |  |  |  |  |  |  |  |  |  |  |  |  |  |  |  |  |  |  |  |  |  |  |  |  |  |  |  |  |  |  |  |  |  |  |  |  |  |  |  |  |  |  |  |  |  |  |  |  |  |  |  |  |  |  |  |  |  |  |  |  |  |  |  |  |  |  |  |  |  |  |  |  |  |  |  |  |  |  |  |  |  |  |  |  |  |  |  |  |  |  |  |  |  |  |  |  |  |  |  |  |  |  |  |  |  |  |  |  |  |  |  |  |  |  |  |  |  |  |  |  |  |  |  |  |  |  |  |  |  |  |  |  |  |  |  |  |  |  |  |  |  |  |  |  |  |  |  |  |  |  |  |  |
|-----------|------------|----|----|----|----|----|----|--------------|-------------|----|-----|--|--------------|--------------|--|--|--|--|--|----------------|--|--|--|--|--|--|--|--|--|--|--|--|--|--|--|--|--|--|--|--|--|--|--|--|--|--|--|--|--|--|--|--|--|--|--|--|--|--|--|--|--|--|--|--|--|--|--|--|--|--|--|--|--|--|--|--|--|--|--|--|--|--|--|--|--|--|--|--|--|--|--|--|--|--|--|--|--|--|--|--|--|--|--|--|--|--|--|--|--|--|--|--|--|--|--|--|--|--|--|--|--|--|--|--|--|--|--|--|--|--|--|--|--|--|--|--|--|--|--|--|--|--|--|--|--|--|--|--|--|--|--|--|--|--|--|--|--|--|--|--|--|--|--|--|--|--|--|--|--|--|--|--|--|--|--|--|--|--|--|--|--|--|--|--|--|--|--|--|--|--|--|--|--|--|--|--|--|--|--|--|--|--|--|--|--|--|--|--|--|--|--|--|--|--|--|--|--|--|--|--|--|--|--|--|--|--|--|--|--|--|--|--|--|--|--|--|--|--|--|--|--|--|--|--|--|--|--|--|--|--|--|--|--|--|--|--|--|--|--|--|--|--|--|--|--|--|--|--|--|--|--|--|--|--|--|--|--|--|--|--|--|--|--|--|--|--|--|--|--|--|--|--|--|--|--|--|--|--|--|--|--|--|--|--|--|--|--|--|--|--|--|--|--|--|--|--|--|--|--|--|--|--|--|--|--|--|--|--|--|--|--|--|--|--|--|--|--|--|--|--|--|--|--|--|--|--|--|--|--|--|--|--|--|--|--|--|--|--|--|--|--|--|--|--|--|--|--|--|--|--|--|--|--|--|--|--|--|--|--|--|--|--|--|--|--|--|--|--|--|--|--|--|--|--|--|--|--|--|--|--|--|--|--|--|--|--|--|--|--|--|--|--|--|--|--|--|--|--|--|--|--|--|--|--|--|--|--|--|--|--|--|--|--|--|--|--|--|--|--|--|--|--|--|--|--|--|--|--|--|--|--|--|--|--|--|--|--|--|--|--|--|--|--|--|--|--|--|--|--|--|--|--|--|--|--|--|--|--|--|--|--|--|--|--|--|--|--|--|--|--|--|--|--|--|--|--|--|--|--|--|--|--|--|--|--|--|--|--|--|--|--|--|--|--|--|--|--|--|--|--|--|--|--|--|--|--|--|--|--|--|--|--|--|--|--|--|--|--|--|--|--|--|--|--|--|--|--|--|--|--|--|--|--|--|--|--|--|--|--|--|--|--|--|--|--|--|--|--|--|--|--|--|--|--|--|--|--|--|--|--|--|--|--|--|--|--|--|--|--|--|--|--|--|--|--|--|--|--|--|--|--|--|--|--|--|--|--|--|--|--|--|--|--|--|--|--|--|--|--|--|--|--|--|--|--|--|--|--|--|--|--|--|--|--|--|--|--|--|--|--|--|--|--|--|--|--|--|--|--|--|--|--|--|--|--|--|--|--|--|--|--|--|--|--|--|--|--|--|--|--|--|--|--|--|--|--|--|--|--|--|--|--|--|--|--|--|--|--|--|--|--|--|--|--|--|--|--|--|--|--|--|--|--|--|--|--|--|--|--|--|--|--|--|--|--|--|--|--|--|--|--|--|--|--|--|--|--|--|--|--|--|--|--|--|--|--|--|--|--|--|--|--|--|--|--|--|--|--|--|--|--|--|--|--|--|--|--|--|--|--|--|--|--|--|--|--|--|--|--|--|--|--|--|--|--|--|--|--|--|--|--|--|--|--|--|--|--|--|--|--|--|--|--|--|--|--|--|--|--|--|--|--|--|--|--|--|--|--|--|--|--|--|--|--|--|--|--|--|--|--|--|--|--|--|--|--|--|--|--|--|--|--|--|--|--|--|--|--|--|--|--|--|--|--|--|--|--|--|--|--|--|--|--|--|--|--|--|--|--|--|--|--|--|--|--|--|--|--|--|--|--|--|--|--|--|--|--|--|--|--|--|--|--|--|--|--|--|--|--|--|--|--|--|--|--|--|--|--|--|--|--|--|--|--|--|--|--|--|--|--|--|--|--|--|--|--|--|--|--|--|--|--|--|--|--|--|--|--|--|--|--|--|--|--|--|--|--|--|--|--|--|--|--|--|--|--|--|--|--|--|--|--|--|--|--|--|--|--|--|--|--|--|--|--|--|--|--|--|--|--|--|--|--|--|--|--|--|--|--|--|--|--|--|--|--|--|--|--|--|--|--|--|--|--|--|--|--|--|--|--|--|--|--|--|--|--|--|--|--|--|--|--|--|--|--|--|--|--|--|--|--|--|--|--|--|--|--|--|--|--|--|--|--|--|--|--|--|--|--|--|--|--|--|--|--|--|--|--|--|--|--|--|--|--|--|--|--|--|--|--|--|--|--|--|--|--|--|--|--|--|--|--|--|--|--|--|--|--|--|--|--|--|--|--|--|--|--|--|--|--|--|--|--|--|--|--|--|--|--|--|--|--|--|--|--|--|--|--|--|--|--|--|--|--|--|--|--|--|--|--|--|--|--|--|--|--|--|--|--|--|--|--|--|--|--|--|--|--|--|--|--|--|--|--|--|--|--|--|--|--|--|--|--|--|--|--|--|--|--|--|--|--|--|--|--|--|--|--|--|--|--|--|--|--|--|--|--|--|--|--|--|--|--|--|--|--|--|--|--|--|--|--|--|--|--|--|--|--|--|--|--|--|--|--|--|--|--|--|--|--|--|--|--|--|--|--|--|--|--|--|--|--|--|--|--|--|--|--|--|--|--|--|--|--|--|--|--|--|--|--|--|--|--|--|--|--|--|--|--|--|--|--|--|--|--|--|--|--|--|--|--|--|--|--|--|--|--|--|--|--|--|--|--|--|--|--|--|--|--|--|--|--|--|--|--|--|--|--|--|--|--|--|--|--|--|--|--|--|--|--|--|--|--|--|--|--|--|--|--|--|--|--|--|--|--|--|--|--|--|--|--|--|--|--|--|--|--|--|--|--|--|--|--|--|--|--|--|--|--|--|--|--|--|--|--|
|           | 1          | 10 | 20 | 30 | 40 | 50 | 60 | 70           | 80          | 90 | 100 |  |              |              |  |  |  |  |  |                |  |  |  |  |  |  |  |  |  |  |  |  |  |  |  |  |  |  |  |  |  |  |  |  |  |  |  |  |  |  |  |  |  |  |  |  |  |  |  |  |  |  |  |  |  |  |  |  |  |  |  |  |  |  |  |  |  |  |  |  |  |  |  |  |  |  |  |  |  |  |  |  |  |  |  |  |  |  |  |  |  |  |  |  |  |  |  |  |  |  |  |  |  |  |  |  |  |  |  |  |  |  |  |  |  |  |  |  |  |  |  |  |  |  |  |  |  |  |  |  |  |  |  |  |  |  |  |  |  |  |  |  |  |  |  |  |  |  |  |  |  |  |  |  |  |  |  |  |  |  |  |  |  |  |  |  |  |  |  |  |  |  |  |  |  |  |  |  |  |  |  |  |  |  |  |  |  |  |  |  |  |  |  |  |  |  |  |  |  |  |  |  |  |  |  |  |  |  |  |  |  |  |  |  |  |  |  |  |  |  |  |  |  |  |  |  |  |  |  |  |  |  |  |  |  |  |  |  |  |  |  |  |  |  |  |  |  |  |  |  |  |  |  |  |  |  |  |  |  |  |  |  |  |  |  |  |  |  |  |  |  |  |  |  |  |  |  |  |  |  |  |  |  |  |  |  |  |  |  |  |  |  |  |  |  |  |  |  |  |  |  |  |  |  |  |  |  |  |  |  |  |  |  |  |  |  |  |  |  |  |  |  |  |  |  |  |  |  |  |  |  |  |  |  |  |  |  |  |  |  |  |  |  |  |  |  |  |  |  |  |  |  |  |  |  |  |  |  |  |  |  |  |  |  |  |  |  |  |  |  |  |  |  |  |  |  |  |  |  |  |  |  |  |  |  |  |  |  |  |  |  |  |  |  |  |  |  |  |  |  |  |  |  |  |  |  |  |  |  |  |  |  |  |  |  |  |  |  |  |  |  |  |  |  |  |  |  |  |  |  |  |  |  |  |  |  |  |  |  |  |  |  |  |  |  |  |  |  |  |  |  |  |  |  |  |  |  |  |  |  |  |  |  |  |  |  |  |  |  |  |  |  |  |  |  |  |  |  |  |  |  |  |  |  |  |  |  |  |  |  |  |  |  |  |  |  |  |  |  |  |  |  |  |  |  |  |  |  |  |  |  |  |  |  |  |  |  |  |  |  |  |  |  |  |  |  |  |  |  |  |  |  |  |  |  |  |  |  |  |  |  |  |  |  |  |  |  |  |  |  |  |  |  |  |  |  |  |  |  |  |  |  |  |  |  |  |  |  |  |  |  |  |  |  |  |  |  |  |  |  |  |  |  |  |  |  |  |  |  |  |  |  |  |  |  |  |  |  |  |  |  |  |  |  |  |  |  |  |  |  |  |  |  |  |  |  |  |  |  |  |  |  |  |  |  |  |  |  |  |  |  |  |  |  |  |  |  |  |  |  |  |  |  |  |  |  |  |  |  |  |  |  |  |  |  |  |  |  |  |  |  |  |  |  |  |  |  |  |  |  |  |  |  |  |  |  |  |  |  |  |  |  |  |  |  |  |  |  |  |  |  |  |  |  |  |  |  |  |  |  |  |  |  |  |  |  |  |  |  |  |  |  |  |  |  |  |  |  |  |  |  |  |  |  |  |  |  |  |  |  |  |  |  |  |  |  |  |  |  |  |  |  |  |  |  |  |  |  |  |  |  |  |  |  |  |  |  |  |  |  |  |  |  |  |  |  |  |  |  |  |  |  |  |  |  |  |  |  |  |  |  |  |  |  |  |  |  |  |  |  |  |  |  |  |  |  |  |  |  |  |  |  |  |  |  |  |  |  |  |  |  |  |  |  |  |  |  |  |  |  |  |  |  |  |  |  |  |  |  |  |  |  |  |  |  |  |  |  |  |  |  |  |  |  |  |  |  |  |  |  |  |  |  |  |  |  |  |  |  |  |  |  |  |  |  |  |  |  |  |  |  |  |  |  |  |  |  |  |  |  |  |  |  |  |  |  |  |  |  |  |  |  |  |  |  |  |  |  |  |  |  |  |  |  |  |  |  |  |  |  |  |  |  |  |  |  |  |  |  |  |  |  |  |  |  |  |  |  |  |  |  |  |  |  |  |  |  |  |  |  |  |  |  |  |  |  |  |  |  |  |  |  |  |  |  |  |  |  |  |  |  |  |  |  |  |  |  |  |  |  |  |  |  |  |  |  |  |  |  |  |  |  |  |  |  |  |  |  |  |  |  |  |  |  |  |  |  |  |  |  |  |  |  |  |  |  |  |  |  |  |  |  |  |  |  |  |  |  |  |  |  |  |  |  |  |  |  |  |  |  |  |  |  |  |  |  |  |  |  |  |  |  |  |  |  |  |  |  |  |  |  |  |  |  |  |  |  |  |  |  |  |  |  |  |  |  |  |  |  |  |  |  |  |  |  |  |  |  |  |  |  |  |  |  |  |  |  |  |  |  |  |  |  |  |  |  |  |  |  |  |  |  |  |  |  |  |  |  |  |  |  |  |  |  |  |  |  |  |  |  |  |  |  |  |  |  |  |  |  |  |  |  |  |  |  |  |  |  |  |  |  |  |  |  |  |  |  |  |  |  |  |  |  |  |  |  |  |  |  |  |  |  |  |  |  |  |  |  |  |  |  |  |  |  |  |  |  |  |  |  |  |  |  |  |  |  |  |  |  |  |  |  |  |  |  |  |  |  |  |  |  |  |  |  |  |  |  |  |  |  |  |  |  |  |  |  |  |  |  |  |  |  |  |  |  |  |  |  |  |  |  |  |  |  |  |  |  |  |  |  |  |  |  |  |  |  |  |  |  |  |  |  |  |  |  |  |  |  |  |  |  |  |  |  |  |  |  |  |  |  |  |  |  |  |  |  |  |  |  |  |  |  |  |  |  |  |  |  |  |  |  |  |  |  |  |  |  |  |  |  |  |  |  |  |  |  |  |  |  |  |  |  |  |  |  |  |  |  |  |  |  |  |  |  |  |  |  |  |  |  |  |  |  |  |  |

Supplement: Additional file 8 — IGH locus diagram with location of the VH genes from O. latipes. All genes were localized as indicated in material and methods (top left). Alignment of aminoacid sequences deduced from VH genes are at the bottom. The alignment was performed and scored according to recommendations of the IMGT. Naming was done using nomenclature proposed by the same organization. Phylogenetic tree of the VH regions from O. latipes is shown on the top right hand side. MEGA4 software, minimum evolution algorithm and JTT matrix were used to draw the tree. Differences by site are activated with gamma parameter 2.5. [file 1471-2148-11-165-S8.PDF]
