# Supplementary material for: Immunoglobulin heavy chains in medaka (Oryzias latipes)
Source: BMC Evol Biol. 2011 Jun 15;11:165. doi: 10.1186/1471-2148-11-165 (PMC3141427; doi:10.1186/1471-2148-11-165)
Supplement: Additional file 4 — Amino acid sequence alignment of medaka IgM. Alignment is made with the nearest sequences found in Genbank. Canonical cysteines are linked by a line. The first cysteine is marked with an arrow and corresponds to the cysteine required for binding to light chains. Other interesting non-canonical cysteines are also marked with an arrow. [file 1471-2148-11-165-S4.PDF]

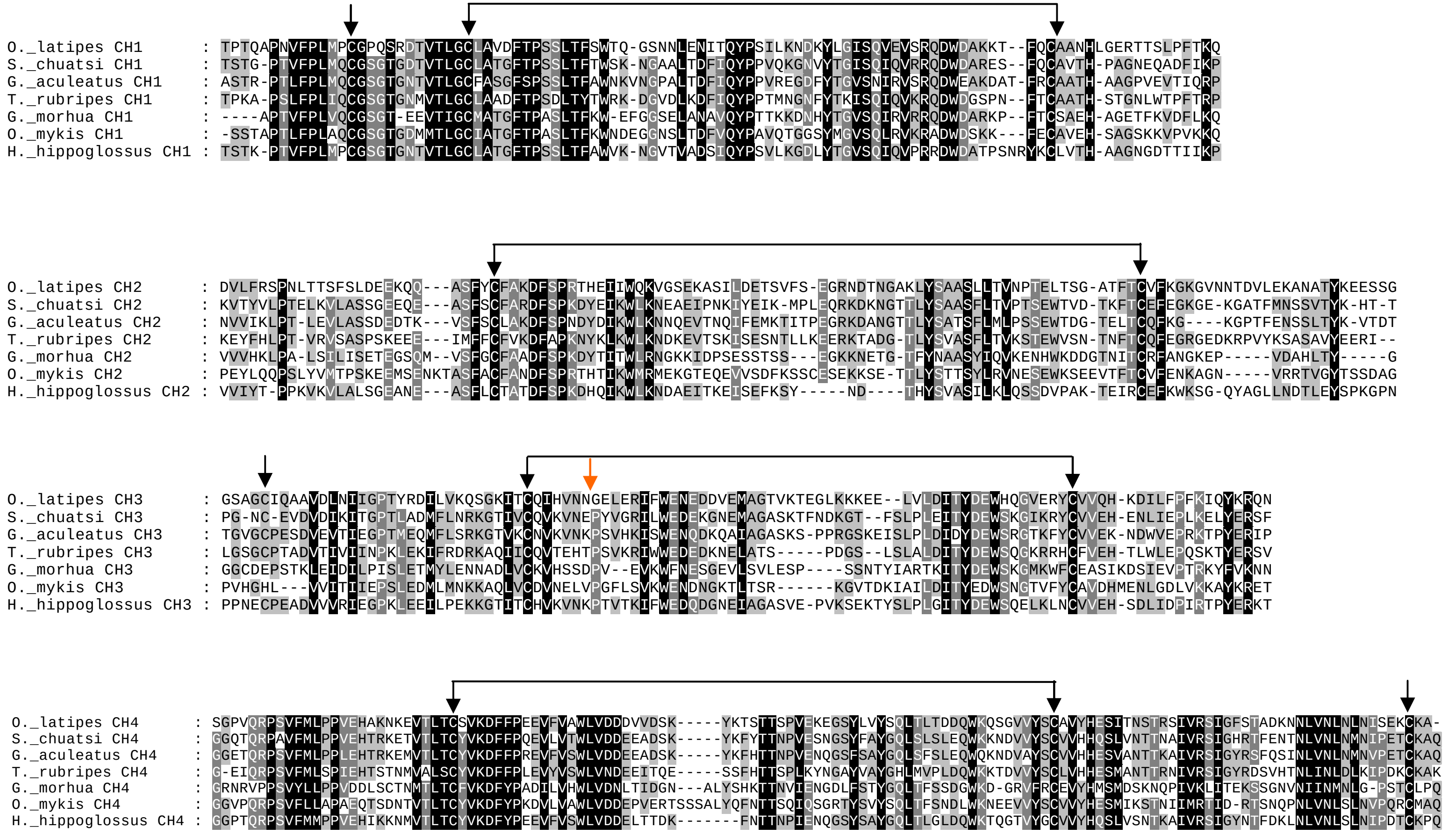

O._latipes CH1 : TPTQAPNVFPLMPCGPQSRDTVTLGCLAVDFTPSSLTFSWTQ-GSNNLENITQYPSILKNDKYLGISQVEVSRQDWDAKKT--FQCAANHLGERTTSLPFTKQ
S._chuatsi CH1 : TSTG-PTVFPLMQCGSGTGDTVTLGCLATGFTPSSLTFTWSK-NGAALTDFIQYPPVQKGNVYTGISQIQVRRQDWDARES--FQCAVTH-PAGNEQADFIKP
G._aculeatus CH1 : ASTR-PTLFPLMQCGSGTGNTVTLGCFASGFSPSSLTFAWNKVNGPALTDFIQYPPVREGDFYTGVSNIRVSRQDWEAKDAT-FRCAATH-AAGPVEVTIQRP
T._rubripes CH1 : TPKA-PSLFPLIQCGSGTGNMVTLGCLAADFTPSDLTYTWRK-DGVDLKDFIQYPPTMNGNFYTKISQIQVKRQDWDGSPN--FTCAATH-STGNLWTPFTRP
G._morhua CH1 : ----APTVFPLVQCGSGT-EEVTIGCMATGFTPASLTFKW-EFGGSELANAVQYPTTKKDNHYTGVSQIRVRRQDWDARKP--FTCSAEH-AGETFKVDFLKQ
O._mykis CH1 : -SSTAPTLFPLAQCGSGTGDMMTLGCIATGFTPASLTFKWNDEGGNSLTDFVQYPAVQTGGSYMGVSQLRVKRADWDSKK---FECAVEH-SAGSKKVPVKKQ
H._hippoglossus CH1 : TSTK-PTVFPLMPCGSGTGNTVTLGCLATGFTPSSLTFAWVK-NGVTVADSIQYPSVLKGDLYTGVSQIQVPRRDWDATPSNRYKCLVTH-AAGNGDTTIIKP
O._latipes CH2 : DVLFRSPNLTTSFSLDEEKQQ---ASFYCFAKDFSPRTHEIIWQKVGSEKASILDETSVFS-EGRNDTNGAKLYSAASLLTVNPTELTSG-ATFTCVFKGKGVNNTDVLEKANATYKEESSG
S._chuatsi CH2 : KVTYVLPTELKVLASSGEEQE---ASFSCFARDFSPKDYEIKWLKNEAEIPNKIYEIK-MPLEQRKDKNGTTLYSAASFLTVPTSEWTVD-TKFTCEFEGKGE-KGATFMNSSVTYK-HT-T
G._aculeatus CH2 : NVVIKLPT-LEVLASSDEDTK---VSFSCLAKDFSPNDYDIKWLKNNQEVTNQIFEMKTITPEGRKDANGTTLYSATSFLMLPSSEWTDG-TELTCQFKG----KGPTFENSSLTYK-VTDT
T._rubripes CH2 : KEYFHLPT-VRVSASPSKEEE---IMFFCFVKDFAPKNYKLKWLKNDKEVTSKISESNTLLKEERKTADG-TLYSVASFLTVKSTEWVSN-TNFTCQFEGRGEDKRPVYKSASAVYEERI--
G._morhua CH2 : VVVHKLPA-LSILISETEGSQM--VSFGCFAADFSPKDYTITWLRNGKKIDPSESSTSS---EGKKNETG-TFYNAASYIQVKENHWKDDGTNITCRFANGKEP-----VDAHLTY-----G
O._mykis CH2 : PEYLQQPSLYVMTPSKEEMSENKTASFACFANDFSPRTHTIKWMRMEKGTEQEVVSDFKSSCESEKKSE-TTLYSTTSYLRVNESEWKSEEVTFTCVFENKAGN-----VRRTVGYTSSDAG
H._hippoglossus CH2 : VVIYT-PPKVKVLALSGEANE---ASFLCTATDFSPKDHQIKWLKNDAEITKEISEFKSY-----ND----THYSVASILKLQSSDVPAK-TEIRCEFKWKSG-QYAGLLNDTLEYSPKGPN
O._latipes CH3 : GSAGCIQAAVDLNIIGPTYRDILVKQSGKITCQIHVNNGELERIFWENEDDVEMAGTVKTEGLKKKEE--LVLDITYDEWHQGVERYCVVQH-KDILFPFKIQYKRQN
S._chuatsi CH3 : PG-NC-EVDVDIKITGPTLADMFLNRKGTIVCQVKVNEPYVGRILWEDEKGNEMAGASKTFNDKGT--FSLPLEITYDEWSKGIKRYCVVEH-ENLIEPLKELYERSF
G._aculeatus CH3 : TGVGCPESDVEVTIEGPTMEQMFLSRKGTVKCNVKVNKPSVHKISWENQDKQAIAGASKS-PPRGSKEISLPLDIDYDEWSRGTKFYCVVEK-NDWVEPRKTPYERIP
T._rubripes CH3 : LGSGCPTADVTIVIINPKLEKIFRDRKAQIICQVTEHTPSVKRIWWEDEDKNELATS-----PDGS--LSLALDITYDEWSQGKRRHCFVEH-TLWLEPQSKTYERSV
G._morhua CH3 : GGCDEPSTKLEIDILPISLETMYLENNADLVCKVHSSDPV--EVKWFNESGEVLSVLESP----SSNTYIARTKITYDEWSKGMKWFCEASIKDSIEVPTRKYFVKNN
O._mykis CH3 : PVHGHL---VVITIIEPSLEDMLMNKKAQLVCDVNELVPGFLSVKWENDNGKTLTSR------KGVTDKIAILDITYEDWSNGTVFYCAVDHMENLGDLVKKAYKRET
H._hippoglossus CH3 : PPNECPEADVVVRIEGPKLEEILPEKKGTITCHVKVNKPTVTKIFWEDQDGNEIAGASVE-PVKSEKTYSLPLGITYDEWSQELKLNCVVEH-SDLIDPIRTPYERKT
O._latipes CH4 : SGPVQRPSVFMLPPVEHAKNKEVTLTCSVKDFFPEEVFVAWLVDDDVVDSK-----YKTSTTSPVEKEGSYLVYSQLTLTDDQWKQSGVVYSCAVYHESITNSTRSIVRSIGFSTADKNNLVNLNLNISEKCKA-
S._chuatsi CH4 : GGQTQRPAVFMLPPVEHTRKETVTLTCYVKDFFPQEVLVTWLVDDEEADSK-----YKFYTTNPVESNGSYFAYGQLSLSLEQWKKNDVVYSCVVHHQSLVNTTNAIVRSIGHRTFENTNLVNLNMNIPETCKAQ
G._aculeatus CH4 : GGETQRPSVFMLPPLEHTRKEMVTLTCYVKDFFPREVFVSWLVDDEEADSK-----YKFHTTNPVENQGSFSAYGQLSFSLEQWQKNDVAYSCVVHHESVANTTKAIVRSIGYRSFQSINLVNLNMNVPETCKAQ
T._rubripes CH4 : G-EIQRPSVFMLSPIEHTSTNMVALSCYVKDFFPLEVYVSWLVNDEEITQE-----SSFHTTSPLKYNGAYVAYGHLMVPLDQWKKTDVVYSCLVHHESMANTTRNIVRSIGYRDSVHTNLINLDLKIPDKCKAK
G._morhua CH4 : GRNRVPPSVYLLPPVDDLSCTNMTLTCFVKDFYPADILVHWLVDNLTIDGN---ALYSHKTTNVIENGDLFSTYGQLTFSSDGWKD-GRVFRCEVYHMSMDSKNQPIVKLITEKSSGNVNIINMNLG-PSTCLPQ
O._mykis CH4 : GGVPQRPSVFLLAPAEQTSDNTVTLTCYVKDFYPKDVLVAWLVDDEPVERTSSSALYQFNTTSQIQSGRTYSVYSQLTFSNDLWKNEEVVYSCVVYHESMIKSTNIIMRTID-RTSNQPNLVNLSLNVPQRCMAQ
H._hippoglossus CH4 : GGPTQRPSVFMMPPVEHIKKNMVTLTCYVKDFYPEEVFVSWLVDDELTTDK-------FNTTNPIENQGSYSAYGQLTLGLDQWKTQGTVYGCVVYHQSLVSNTKAIVRSIGYNTFDKLNLVNLSLNIPDTCKPQ
