# Supplementary material for: Immunoglobulin heavy chains in medaka (Oryzias latipes)
Source: BMC Evol Biol. 2011 Jun 15;11:165. doi: 10.1186/1471-2148-11-165 (PMC3141427; doi:10.1186/1471-2148-11-165)

ZONE 1
(JHs-IGHM)

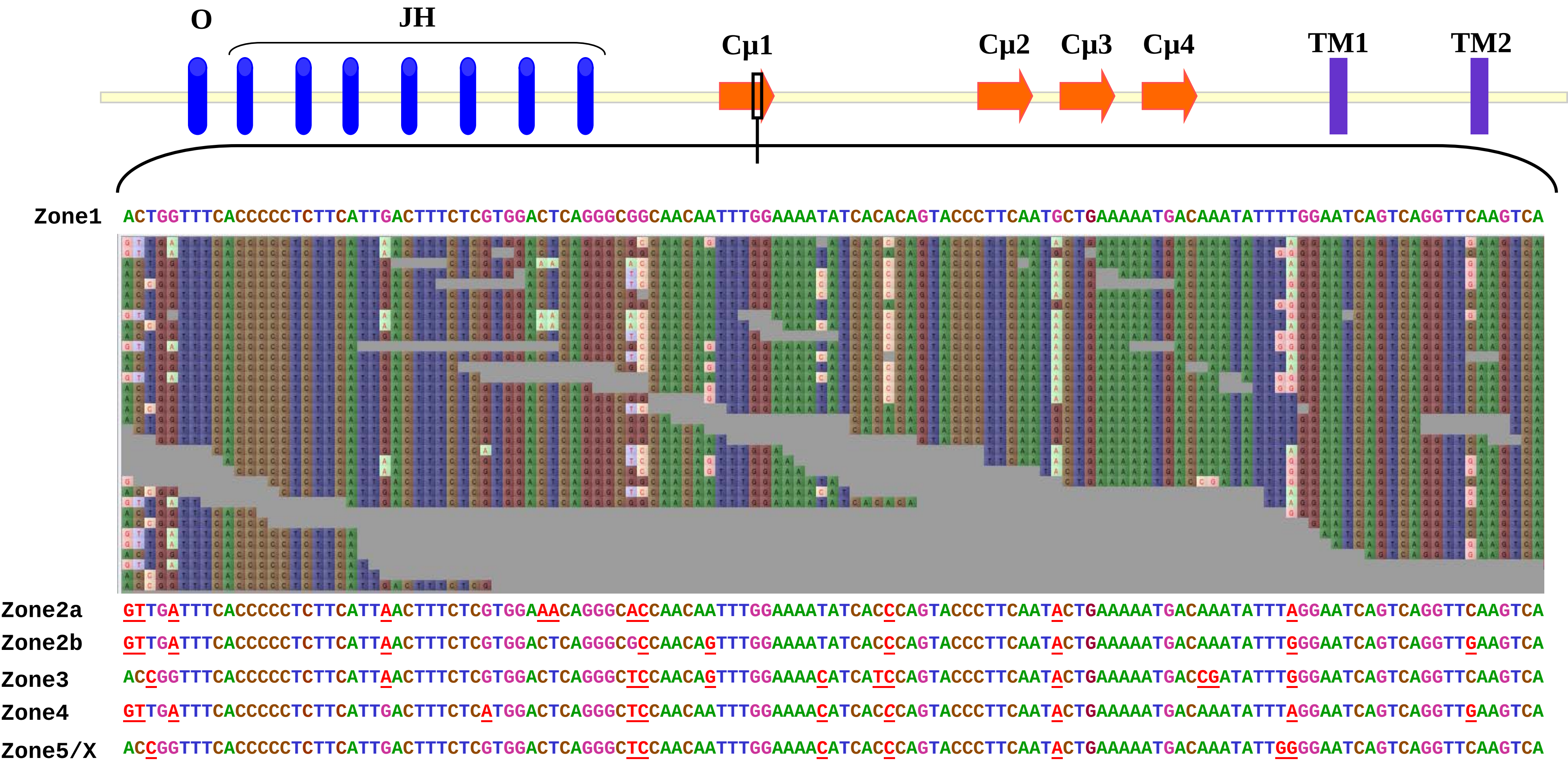

Supplement: Additional file 9 — Confirmation of different genomic zones through genomic short reads alignment. This file shows the result obtained after aligning medaka genomic short reads (DRA000220) with the region of zone 1 that presents JH segments and the IGHM gene. Alignment details of short reads with the Cμ1 are indicated. It includes the specific nucleotide sequence to each zone. Identical nucleotides are shown in the same colour (A: green, C: brown, G: lilac, T: blue) and differences are in red and underlined. [file 1471-2148-11-165-S9.PDF]
